# SNMMI Procedure Standard/EANM Practice Guideline for Fibroblast Activation Protein (FAP) PET

**DOI:** 10.2967/jnumed.124.269002

**Published:** 2025-01

**Authors:** Thomas A. Hope, Jeremie Calais, Ajit H. Goenka, Uwe Haberkorn, Mark Konijnenberg, Jonathan McConathy, Daniela E. Oprea-Lager, Laura Trimnal, Elcin Zan, Ken Herrmann, Christophe M. Deroose

**Affiliations:** 1 Department of Radiology and Biomedical Imaging, University of California San Francisco, San Francisco, California;; 2 UCSF Helen Diller Family Comprehensive Cancer Center, University of California San Francisco, San Francisco, California;; 3 Department of Radiology, San Francisco VA Medical Center, San Francisco, California;; 4 Ahmanson Translational Theranostics Division, Department of Molecular and Medical Pharmacology, David Geffen School of Medicine, University of California Los Angeles, Los Angeles, California;; 5 Jonsson Comprehensive Cancer Center, University of California Los Angeles, Los Angeles, California;; 6 Department of Radiology, Mayo Clinic, Rochester, Minnesota;; 7 Department of Nuclear Medicine, University Hospital Heidelberg, Heidelberg, Germany;; 8 Radiology and Nuclear Medicine Department, Erasmus MC, Rotterdam, Netherlands;; 9 Department of Radiology, University of Alabama at Birmingham, Birmingham, Alabama;; 10 Department of Medical Imaging, Radboud University Medical Center, Nijmegen, The Netherlands;; 11 Department of Radiology, Cleveland Clinic, Cleveland, Ohio;; 12 Department of Nuclear Medicine, University Hospital Essen, University of Duisburg–Essen, Essen, Germany;; 13 German Cancer Consortium, Partner Site University Hospital Essen, and German Cancer Research Center, Essen, Germany;; 14 Nuclear Medicine, University Hospitals Leuven, Leuven, Belgium; and; 15 Nuclear Medicine and Molecular Imaging, Department of Imaging and Pathology, KU Leuven, Leuven, Belgium

## 1. PREAMBLE

The Society of Nuclear Medicine and Molecular Imaging (SNMMI) is an international scientific and professional organization founded in 1954 to promote the science, technology, and practical application of nuclear medicine. The European Association of Nuclear Medicine (EANM) is a professional non-profit medical association that facilitates communication worldwide between individuals pursuing clinical and research excellence in nuclear medicine. The EANM was founded in 1985. SNMMI and EANM members are physicians, technologists, and scientists specializing in the research and practice of nuclear medicine.

The SNMMI and EANM will periodically define new guidelines for nuclear medicine practice to help advance the science of nuclear medicine and to improve the quality of service to patients throughout the world. Existing practice guidelines will be reviewed for revision or renewal, as appropriate, on their fifth anniversary or sooner, if indicated.

Each practice guideline, representing a policy statement by the SNMMI/EANM, has undergone a thorough consensus process in which it has been subjected to extensive review. The SNMMI and EANM recognize that the safe and effective use of diagnostic nuclear medicine imaging requires specific training, skills, and techniques, as described in each document. Reproduction or modification of the published practice guideline by those entities not providing these services is not authorized.

These guidelines are an educational tool designed to assist practitioners in providing appropriate care for patients. They are not inflexible rules or requirements of practice and are not intended, nor should they be used, to establish a legal standard of care. For these reasons and those set forth below, both the SNMMI and the EANM caution against the use of these guidelines in litigation in which the clinical decisions of a practitioner are called into question.

The ultimate judgment regarding the propriety of any specific procedure or course of action must be made by the physician or medical physicist in light of all the circumstances presented. Thus, there is no implication that an approach differing from the guidelines, standing alone, is below the standard of care. To the contrary, a conscientious practitioner may responsibly adopt a course of action different from that set forth in the guidelines when, in the reasonable judgment of the practitioner, such course of action is indicated by the condition of the patient, limitations of available resources, or advances in knowledge or technology subsequent to publication of the guidelines.

The practice of medicine includes both the art and the science of the prevention, diagnosis, alleviation, and treatment of disease. The variety and complexity of human conditions make it impossible to always reach the most appropriate diagnosis or to predict with certainty a particular response to treatment.

Therefore, it should be recognized that adherence to these guidelines will not ensure an accurate diagnosis or a successful outcome. All that should be expected is that the practitioner will follow a reasonable course of action based on current knowledge, available resources, and the needs of the patient to deliver effective and safe medical care. The sole purpose of these guidelines is to assist practitioners in achieving this objective.

## 2. INTRODUCTION

Fibroblast activation protein (FAP) is a transmembrane protein expressed on activated fibroblasts that functions as a serine protease (1). FAP is part of a family of peptidases with family members including dipeptidyl peptidase IV (DPP4) and prolyl oligopeptidase (PREP) (2). It is expressed on both cancer-associated fibroblasts (CAFs) and normal activated fibroblasts (NAFs) involved in wound healing and tissue repair (3). FAP has long been a target for cancer therapy (4), but the development of FAP targeted radioligands has led to an increased interest in imaging FAP for assessment of cancer and other diseases (5). Although the FAP PET will play a role in non-oncologic diseases, the primary focus of this guideline is its oncologic applications. Due to the relatively wider use of ^68^Ga-FAPI-04 and other quinoline-based radiopharmaceuticals, clinical results and recommendations in this guideline were obtained primarily based on this family of radiopharmaceuticals.

## 3. GOALS

The goal of providing guidelines is to assist providers in recommending, performing, interpreting and reporting the results of FAP PET imaging studies. This document aims to provide referring providers with the best available evidence, to inform where robust evidence is lacking, and to help them to deliver the best possible diagnostic efficacy and study quality for their patients. This guideline also presents standardized quality control/quality assurance (QC/QA) procedures and imaging procedures for FAP PET. Adequate precision, accuracy, repeatability, and reproducibility are essential for the clinical management of patients and the use of FAP PET within multicenter trials. A standardized imaging procedure will help to promote the appropriate use of FAP PET and enhance subsequent research.

## 4. POTENTIAL CLINICAL INDICATIONS

FAP-targeted PET offers a new approach in molecular imaging for oncological and non-oncological diseases, though its full clinical applications are yet to be determined. Tumoral stroma can make up 90% of the volume of a tumor, making stroma detection by molecular imaging potentially a better strategy than direct detection of the malignant cells. Additionally, FAP expression increases in fibroblasts activated in multiple remodeling processes, such as wound healing, inflammation, and fibrosis. Thus, FAP PET has the potential to be used in both oncological and non-oncological applications. In both cases, FAP PET imaging can be used for initial staging, re-staging, therapy response evaluation and whole-body target expression assessment for therapy selection. However, currently, there are no approved clinical indications for FAP PET. The indications proposed below are only potential or promising applications inferred from the current literature and ongoing clinical trials (6).

### Oncology

There are three categories of tumors in the context of FAP imaging: desmoplastic tumors that have a high concentration of CAFs, tumors that do not have a significant desmoplastic reaction, and tumors where FAP is expressed on both the tumor stroma and the tumor cells.

Tumors that have desmoplastic reaction and, therefore, a high content of FAP-expressing CAFs, include gastro-intestinal adenocarcinoma, pancreatic ductal adenocarcinoma (PDAC), cholangiocarcinoma, esophageal, head and neck cancer, thyroid, cancer of unknown primary (CUP), lung, peritoneal, bladder, ovarian and breast cancers. For instance, in PDAC, the desmoplastic stroma makes up 60-70% of the tumor volume and prominently features FAP-expressing CAFs that influence fibrosis, tumor spread, and treatment resistance (7). In lung cancer, FAP PET has been shown to benefit N-staging and M-staging, particularly in pleural, liver and brain metastasis (8). In breast cancer, there is increased FAP ligand uptake that is independent of histological phenotype (lobular or ductal) and molecular subtype according to hormone receptor expression and human epidermal growth factor receptor 2 expression (9,10).

There is particular interest for using FAP PET in settings where physiologic uptake on FDG PET limits diagnostic utility and in tumors with low FDG avidity. Metastatic brain tumors are shown to have increased uptake on FAP PET, although there is currently no evidence demonstrating a benefit of FAP PET compared to standard modalities for the evaluation of primary brain tumors. In head and neck squamous cell carcinoma, FAP PET appears to reduce false positive results obtained by FDG with respect to regional nodal metastases (11). One of the main regions with decreased physiologic uptake could be in peritoneal imaging, where bowel activity limits FDG PET, and this has been shown to be beneficial in ovarian cancer and gastric cancer (12,13).

On the other hand, several cancer types do not induce a strong and/or consistent FAP uptake such as lymphoma, myeloma, prostate adenocarcinoma, renal cell carcinoma, melanoma and seminoma. It is unlikely that FAP PET will play a significant role in staging of these cancers. Lastly, tumors of mesenchymal origin express FAP on both CAFs and tumor cells, which is of particular interest in sarcomas. Although sarcomas can have high uptake on FAP PET, it does not appear to improve staging compared to FDG PET, and its role may be limited to sarcomas with low FDG avidity and high FAP expression (e.g. solitary fibrous tumor) and selection for radioligand therapy (RLT) (14).

Evaluating the use of FAP PET for treatment response is in its early stages, but early studies suggest that FAP PET can accurately measure response to treatment (15,16). Although treatment-induced fibrosis, inflammation and necrosis could represent challenges for this indication, this may be limited to treatments that induce fibrotic responses such as external beam radiation therapy. Additionally, surgery can result in fibrosis seen on FAP PET that can persist for up to 8 months (17). With the future approval of FAP targeted therapies, both RLTs and non-RLT therapies, the role of FAP PET as a biomarker for therapeutic target assessment may become important.

### Non-Oncology

As a marker of activated fibroblasts, FAP is a promising biomarker for a range of inflammatory and fibrosing diseases. Preliminary studies in small cohorts of patients have shown increased FAP PET signal in a wide range of settings including cardiac injury, interstitial lung disease and pulmonary fibrosis, IgG4-related disease, cirrhosis, renal injury, inflammatory bowel disease and rheumatoid arthritis (18–21). Several studies suggest that FAP PET is better suited for imaging the fibrotic phase of these disease processes compared to FDG PET. FAP PET may also be suited for monitoring response to therapies that slow or reverse fibrosis.

The relatively low FAP accumulation in most normal tissues is advantageous for whole body imaging of inflammation and fibrosis. Unlike FDG, which has diet-dependent variable myocardial uptake, FAP ligands have low activity in the normal myocardium and cardiac blood pool. FAP PET can detect fibroblast activation and cardiac remodeling after acute myocardial infarction, with a potential predictive role of FAP uptake in the evolution of ventricular dysfunction (22–24). FAP PET may also be useful for detecting fibrosis related to chemotherapy and radiation-induced myocardial injury, heart failure, cardiomyopathy and pulmonary hypertension (25).

Several groups have evaluated the utility of FAP PET for assessing pulmonary fibrosis associated with interstitial lung disease (26,27). FAP PET demonstrates increased signal in fibrotic lung compared to radiographically normal lung, and early data suggest that higher FAP ligand binding correlates with more active and extensive pulmonary fibrosis (27). Additional studies are needed to determine if FAP PET can predict functional and clinical outcomes better than high-resolution chest CT and pulmonary function tests alone. FAP PET also has the potential to predict and monitor response to anti-fibrotic agents, such as nintedanib and pirfenidone that are used clinically to slow down pulmonary fibrosis. Overall, these proof-of-concept studies on diverse non-oncologic diseases have attracted strong interest in this domain. However, the existing data is inadequate to incorporate this research into clinical application, indicating a need for further studies on FAP PET in infectious, inflammatory, and rheumatological conditions.

### Biomarker Concept

FAP is currently explored as a potential target for a number of different FAP-directed therapies. A potential biomarker allowing for visualization and quantitation of FAP expression is urgently needed. Such a biomarker allows to better select and monitor patients undergoing FAP-directed therapies. Whereas FAP-directed RLT is still in its infancy, there are several different mechanisms of action (antibodies, small molecule inhibitors, pro-drugs (NCT04969835) and CAR-T cell therapy (NCT01722149)) in clinical translation (28,29). Pursuing FAP PET as biomarker concept addresses two challenges at the same time: providing evidence to regulators that FAP PET indeed correctly assesses FAP-expression (correlation with immunohistochemistry as gold standard) and offering a valuable tool for selecting and monitoring patients for FAP-targeted therapies.

## 5. QUALIFICATIONS AND RESPONSIBILITIES OF PERSONNEL

### A. Physician

FAP PET examinations should be performed by, or under the supervision of, a physician specialized in nuclear medicine and certified by accrediting boards. Physicians who interpret FAP PET results should also complete appropriate training programs provided by the manufacturers of approved radiotracers.

### B. Technologist

FAP PET examinations should be performed by qualified registered or certified nuclear medicine technologists. See Performance Responsibility and Guidelines for the Nuclear Medicine Technologist for further details (https://snmmi.org/common/Uploaded%20files/Web/Clinical%20Practice/NMT-Scope-of-Practice-and-Performance-Standards-2nd-Ed-2022-Complete-App.pdf). According to location of practice, additional qualifications may be requested for technologists to use the computed tomography (CT) and/or magnetic resonance (MR) component of the scanner.

### C. Medical Physicist

PET systems should comply with the international standard of quality, including dosimetry and radiation protection procedures to limit the radiation exposure of patients and healthcare personnel. A medical physicist should optimize protocols, ensuring that the established standards are met. A medical physicist can assist physicians to adhere to good practice and maintain it, by monitoring and optimizing radiation dose and developing algorithms to reduce the radiation exposure of the CT component.

## 6. PROCEDURE/SPECIFICATIONS OF THE EXAMINATION

### Request

The prescribing physician should provide a written request form providing information about the medical condition of the patient, including relevant medical history and one or more specific clinical questions that the PET should address, allowing for the justification and coding of the examination. Previous medical procedures that can promote fibroblast activity (e.g. surgery, biopsy, radiation therapy) should be mentioned. Information obtained in prior imaging studies should be provided as well. Lesions outside of the classical field-of-view (FOV) of a whole-body PET (e.g. vertex, arms, lower limbs) should be mentioned. Information relevant for the hybrid partner examination (CT or MRI) needs to be provided, including claustrophobia as well as recent renal function (glomerular filtration rate) and history of hypersensitivity reactions to iodinated or gadolinium-containing contrast media for contrast-enhanced CT or MRI, respectively. Confirmation that the patient is not pregnant and ongoing lactation should be mentioned, as well. Currently there are no known drug interactions for FAP ligands. It is useful to mention if a patient is taking fibroblast-targeting drugs such as nintedanib or pirfenidone (30).

### Patient Preparation and Precautions

The patient should be well hydrated to promote clearance of urinary excreted tracer. In contrast to FDG imaging, no caloric fasting nor adaption of anti-diabetic drugs is necessary, as glycemia and insulinemia have no influence on biodistribution and lesion uptake. Avoiding strenuous exercise in the preceding 24 hours is not required.

General radiopharmaceutical administration procedures to handle potential pregnancy and lactation should be applied. It is currently not known if there are detrimental effects of exposure to FAP tracers in utero. In case of documented pregnancy, alternative imaging procedures should be strongly considered. In women of childbearing potential, in case of uncertainty regarding potential pregnancy, point of care testing should be performed according to the PET center’s standard procedure, which can include urinary or serum testing on the day of the examination. Precautions for lactating women depend on radionuclide and injected activity; an interruption of 4 to 24 hours of lactation can be requested, depending on radionuclide and institutional policy.

FAP PET can be considered in pediatric patients, although experience in children is limited (31,32). No adverse events have been reported in these rare cases. Proper procedures for immobilization, adapted to the age of the child and their anticipated compliance, should be available, ranging from restraining devices to sedation to general anesthesia, similar to other PET imaging procedures.

### Radiopharmaceuticals

**FIGURE 1.**
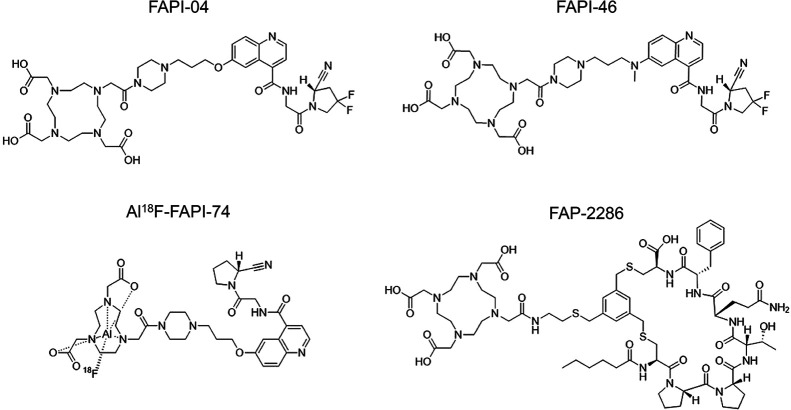
Chemical structures of FAP PET radiopharmaceuticals.

There are innumerable radiopharmaceuticals that have been developed targeting FAP. The most commonly used are quinoline based, but more recently peptide and peptidomimetic compounds have been developed (Fig. 1).

#### A) Quinoline Based Radiopharmaceuticals

The development of selective nanomolar affinity FAP inhibitors based on a (4-quinolinoyl)-glycyl-2-cyanopyrrolidine scaffold, with limited affinity for DPP4 and PREP, paved the way for the development of small molecule FAP tracers. The moiety currently referred to as UAMC1110 emerged as one of the most promising vector moieties.^68^Ga-FAPI-04, containing this quinoline-based UAMC1110 FAP inhibitor coupled to a DOTA chelator by a short linker, was one of the first to demonstrate the potential of FAP PET and has been used in the majority of FAP PET publications (5). Different modifications have been introduced to FAPI-04 resulting in a class of quinoline-based PET tracers, including FAPI-02, FAPI-42, FAPI-46 and FAPI-74. FAPI-46 uses the same vector moiety and DOTA chelator but uses a slightly altered linker. FAPI-42 (same vector moiety as FAPI-04) and FAPI-74 (same vector moiety as FAPI-02) both contain a NOTA chelator, allowing labeling with fluorine-18 using the Al^18^F-radiolabeling method.

There are multiple additional variations on quinoline-based FAP radiopharmaceuticals, focused on increasing binding affinity. For example, the OncoFAP family uses an 8-amido-quinoline and optimized linker, with ^68^GaDOTAGA-OncoFAP as candidate diagnostic PET tracer (33,34). Other optimization strategies of quinoline-based tracers include multimeric compounds, e.g. dimeric FAP ligands such as ^68^Ga-DOTA-2P(FAPI)_2_ (35), which contains 2 UAMC1110 motifs.

Current quinoline-based tracers are characterized by rapid in vivo tumoral uptake (from 10 minutes post injection) but substantial wash-out after 24 hours (36), making them good diagnostic moieties that are less suited for use with therapeutic radionuclides with a long half-life. There is limited direct comparison in humans between different FAP tracers. Presumably, results of the different quinoline-based tracers that have demonstrated a good tracer profile in the clinical setting (high tumor uptake, low background uptake) will be quite similar and can be treated as a class.

#### B) Non-quinoline Based Radiopharmaceuticals

Other FAP targeting molecules have been developed and used as backbone to develop non-quinoline based radiopharmaceuticals. FAP-2286 is based on a seven amino acid cyclic peptide with affinity for FAP in the nanomolar range and includes a DOTA-chelator allowing labeling with ^68^Ga and ^177^Lu (37–39). FAP-2286 has shown limited wash out (∼10%) at 48 hours post-injection, making this an interesting backbone for diagnostic and therapeutic applications. A recent overview on the different stages of clinical development of FAP radioligands has been provided by Mosessian *et al*. (6).

### Administered Activity

The injected activity depends on the radiopharmaceutical used, the radionuclide it contains, the uptake time (interval injection-scan) and the type of camera used (time-of-flight (TOF) versus non-TOF; field-of-view of PET camera). Most studies using gallium-68 based radiopharmaceuticals use administered activities ranging from 120-300 MBq, with average values around 150 MBq, resulting in roughly 2.0 MBq/kg, imaged using 15-20 cm FOV TOF PET cameras. Overall, an administered activity of 100-200 MBq or 2 MBq/kg is recommended for gallium-68 compounds. For fluorine-18 based radiopharmaceuticals, which can typically be produced in higher amounts, the administered activity tends to be higher, with ranges from 185-300 MBq, with average values around 230 MBq, resulting in roughly 4.4 MBq/kg. Based on this experience, an administrated activity of 175-275 MBq or 3-4 MBq/kg is recommended.

Total body PET cameras allow for substantial reduction of the injected activity. One study with ^68^Ga-FAPI-04 obtained good image quality after injection of 0.84-1.14 MBq/kg (around half of advised activity for conventional PET cameras) and a 2 minute imaging time (40). Reducing the above mentioned activities by a factor of 2 to 4 can be envisioned for these cameras.

### Uptake Time

The uptake time usually ranges between 30 and 60 minutes after administration of ^68^Ga-FAPI compounds (41). This time interval was demonstrated to offer a stable, high level of detection rate, regarding both primary tumors and metastatic lesions. Early scan acquisitions, e.g., 10 or 20 minutes after injection, have been reported, and lesion uptake is relatively stable between 20 and 120 minutes (42,43). Late time points 1 h and 3 h after injection have been also proposed. These result in improved discrimination between malignant and chronic inflammatory or fibrotic ^68^Ga-FAPI avid lesions (44). Following clinical applicability and feasibility, acquisitions at 30–40 minutes after injection seem to be a reasonable compromise for ^68^Ga-labeled FAPI tracers (41). For ^18^F-FAPI-74, the recommended uptake time is 60 minutes, resulting in optimal tumor to background ratios with limited background noise (45). Overall, we recommend an uptake time of 20 to 60 minutes for gallium-68 labeled compounds and 30 to 90 minutes for fluorine-18 labeled compounds. Many sites use 60 minute uptake time to match the uptake time with FDG, SSTR and PSMA PET. Of note, the Phase 2 trials of 68Ga-FAPI-46 are using 15-25 minutes uptake time (NCT05262855).

### Image Acquisition

Patients should be instructed to void prior to the scan, to minimize artifacts from bladder activity. PET coverage should be identical to the anatomical CT scan range. The scan range is usually from vertex or skull base to mid-thigh; however, a scan range from skull vertex to toes may be considered, if extending the range coverage would be beneficial for the clinical question. Scanning from mid-thigh to head is recommended to avoid filling of the bladder during scanning.

PET and CT acquisition parameters will be scanner- and institution-dependent. State-of-the-art TOF scanners with improved technology (i.e., enhanced contrast and improved signal-to-noise ratio) may allow for shorter scan times with optimal lesion detection and image quality (46). When using long axial field of view PET scanners, the injected activity of radioactivity may be reduced according to the optimized local protocol (47). PET should be paired with a low-dose CT for attenuation correction and anatomical correlation. A diagnostic CT scan with intravenous contrast may be considered in the same session, following the PET/low-dose CT acquisition. If intravenous CT contrast is used, contrast enhanced CT in the portal venous phase is generally recommended. PET scans are typically acquired in 3D mode with an acquisition time of usually 1–4 minutes per bed position (or equivalent speed using continuous table movement) adjusted to the injected activity (48).

## 7. DOCUMENTATION AND REPORTING

### Study Identification

The final report should include the full name of the patient, sex assigned at birth, medical record number, date of birth, and date of the examination.

### Clinical Information

As a minimum, a summary of relevant clinical history should include reason for referral and the specific clinical question to be answered. If known, the primary location and type of tumor should be noted including relevant prior therapies. The type and date of comparison studies should be stated. If no comparison studies are available, a statement should be made to that effect.

### Technical Details

Study-specific information should include the radiopharmaceutical, the injected activity in megabecquerels (MBq) or millicuries (mCi), the route (intravenous) and anatomic site of administration, and the date and time of administration. If extravasation is seen, it should also be noted. The uptake time (i.e. the interval between the administration of the radiopharmaceutical and the start time of the acquisition) should be reported. The body parts covered by imaging should be described. Any nonstandard position of the patient should be stated.

The direction and range of the acquired images should be stated (i.e., “images were acquired from the vertex to the midthigh”). If a CT was performed for attenuation correction and anatomic registration of the emission images only, the description may be limited to a short statement including the mAs and kVp. If a diagnostic CT was performed, then a more detailed description of the CT protocol and anatomic findings should be provided. Dosimetry parameters should be included if required by national or local regulations. The report should state whether contrast-enhanced or non-enhanced CT was used for attenuation correction.

### Description of Findings

#### Biodistribution

The physiologic biodistribution of most small molecule FAP radiopharmaceuticals in normal organs is rapidly reached within 15 minutes post-injection and only minor changes in biodistribution are seen between 10 minutes and 3 hours (49). The biodistribution of FAP radiopharmaceuticals in normal organs includes, in the decreasing order of uptake: kidney, urinary bladder (excretion), uterus, major salivary glands, pancreas, Waldeyer’s ring, breasts, striated muscles, thyroid, prostate, ovaries, testis, adrenal glands, heart, and blood pool (50). The renal collecting system and urinary bladder are the organs of highest exposure due to urinary excretion of the currently available FAP radiopharmaceuticals. Additionally, some FAP radiopharmaceuticals, such as the Al^18^F-NOTA based ^18^F-FAPI-74, can have biliary excretion and hence high activity concentration in gall bladder, cystic and bile duct (51).

#### General Interpretation

Visual assessment should start with reviewing the maximum intensity projection (MIP) images and axial slices. The inherently low uptake of FAP-targeting radiopharmaceuticals in normal organs allows one to visualize pathologic sites on MIPs. In general, uptake greater than surrounding background that is not attributable to physiologic biodistribution or known non-oncologic causes of uptake is considered malignant. A description of the location and pattern of the uptake should be described (e.g. focal, diffuse or linear).

All lesions should be interpreted considering the full medical history of the patients, including past medical conditions (chronic (fibro)inflammatory or infectious disease, granulomatous disorders, etc.), surgical and medical interventions, clinical status at the time of the scan (acute inflammation, fever, recent procedure) and complemented with a critical appraisal of the corresponding CT (or MRI) findings at that site. In case of lack of corroboration of the malignant nature of a lesion by the morphological imaging and substantial impact on clinical management (e.g. substituting a non-curative approach for an intended curative one or substantial increase of a radiation treatment plan), histological confirmation or dedicated imaging is warranted.

One of the main issues with FAP PET is heterogeneity in expression. Unlike with FDG PET, where uptake is typically correlated with aggressiveness, FAP PET uptake may be due to many factors, including increased cancer cell migration, epithelial-mesenchymal transition, immunosuppression, promotion of angiogenesis, and chemotherapy/immunotherapy resistance. Additionally, since CAFs can have varying origins (mesenchymal stem cells, epithelial cells, adipocytes, preadipocytes, resident fibroblast, endothelial cells and others), it is difficult to use a unique marker which can be used for the identification of all CAFs (52). This heterogeneity not only impacts imaging, but will also interfere with the efficacy of any FAP-related RLT.

#### Semi-Quantitative Analysis

Quantification of uptake using the standardized uptake value (SUV) may not be reproducible across scanners and institutions without standardized acquisition protocols, phantom-based scanner qualifications and cross-calibrations for the specific radionuclide used (gallium-68, fluorine-18). In addition, SUV can be affected by lesion size and to a lesser extent uptake time, with less effect seen than in other tracers such as FDG (43). Given the absence of acceptable reference organs, a qualitative uptake scale such as mild, moderate, intense has not been defined. Changes in SUV (increase or decrease) have not yet been proven to correlate with treatment response. One would expect to see complete normalization or a decrease of tracer uptake as a potential indicator of treatment response, as has been documented in neoadjuvant breast cancer treatment (16), although treatment may induce fibrosis which can lead to FAP uptake that confounds interpretation of treatment response.

#### Incidental Findings, Normal Variants and Important Pitfalls

There are numerous causes of non-oncological uptake on FAP PET, which are important to know when interpreting FAP PET (Table 1) (53–55). In one series, 80% of patients imaged using ^68^Ga-FAPI-04 PET had uptake in benign lesions (55), with musculoskeletal findings being the most common including osteoarthritis, exostosis, and enthesopathy. Non-malignant uptake in hormone-responsive organs needs to be recognized to accurately interpret FAP PET scans. Studies have shown elevated FAP ligand uptake in the uterus of women of reproductive age, which is less prominent in women post-menopausal (56). Radiopharmaceutical uptake in the breast and ovaries was found also to be higher in pre-menopausal than postmenopausal women (57).

**TABLE 1.** Non-oncologic and Common Pitfalls Seen with FAP PET

Benign lesions
Focal nodular hyperplasia
Hemorrhoids
Splenic hemangioma
Thyroid adenoma
Fibrotic processes
Cardiac fibrosis
Hepatic fibrosis
Pulmonary fibrosis
Myelofibrosis
Wound healing
Inflammatory
Atherosclerosis/arteritis
Esophagitis
IgG4-related processes
Inflammatory bowel disease
Pancreatitis
Periodontitis
Pneumonia
Tuberculosis
Musculoskeletal lesions
Avascular necrosis
Degenerative changes
Enthesopathy
Exostosis
Fracture
Schmorl’s nodes
Arthritis
Physiologic organ uptake
Mammary tissue
Pancreatic
Uterine
Ovaries
Gall bladder

Nonmalignant findings in patients with fibrotic processes will be detected by FAP and should be kept in mind during image interpretation. For example, non-oncologic uptake has been reported in Ig-G4-related disease (58), in muscle and wound healing (59), and in diseases associated with a fibrotic reaction (e.g., myelofibrosis, granulomatous disease and liver cirrhosis) (60).

In patients with pancreatic cancer, uptake distal to the primary tumor can be caused by (retro-obstructive) inflammation and may sometimes obscure tumor boundaries. Dynamic and delayed scanning has been proposed to distinguish benign from malignant uptake, but currently cannot be recommended although it is an area for further study (61–63). However, the pattern of FAP uptake is critically important as PDAC can present with diffuse pancreatic uptake due to pancreatic inflammation and may lead to over-staging of the primary tumor (44).

## 8. DOSIMETRY

Radiation dosimetry by the different FAP PET radiopharmaceuticals is similar (Table 2), with the highest absorbed doses in the urinary bladder wall (median 0.048 mGy/MBq) and kidneys (median 0.016 mGy/MBq). The median effective dose for the ^68^Ga-labeled FAP radiopharmaceuticals is 0.0123 mSv/MBq and for ^18^F-FAPI-74 it is 0.0141 mSv/MBq. When using an activity of 100-200 MBq ^68^Ga FAP the effective dose will be in the range of 1.0-2.5 mSv and with 185-300 MBq ^18^F FAPI-74 the effective dose will range 2.6-4.2 mSv. Both ranges are comparable to the effective doses encountered with ^68^Ga-PSMA-11 and ^18^F-DCFPyL. Absorbed dose by the CT scan is not included in this dose value as it depends on the protocol (diagnostic or attenuation correction) and the CT hardware. When only attenuation correction is needed from the CT data significant reduction in CT dose is achievable to 1 mSv.

**TABLE 2.** Dosimetry for ^68^Ga-FAPI Based on 4 Studies (N=18), for ^18^F-FAPI-74 Based on a Single Study (N=10), and for ^68^Ga-FAP-2286 Based on a Single Study (N=6)

Organ dosimetry (mGy/MBq)	[^68^Ga]Ga-FAPI-46 (n=6)	[^68^Ga]Ga-FAPI-RGD (n=6)	[^68^Ga]Ga-FAPI-04 (n=6)	[^18^F]F-FAPI-74 (n=10)	[68Ga]Ga-FAP-2286 (n=6)
Reference	Meijer 2020	Zang 2022	Wang 2021	Giesel 2021	Kline 2024
Gallbladder wall	0.0056 ± 0.0009	0.0084 ± 0.0002	< 0.0001	0.0117 ± 0.0010	0.0098 ± 0.0027
Lower large intestine wall	0.0057 ± 0.0007	0.0078 ± 0.0005	0.0003 ± 0.0001	0.0123 ± 0.0016	0.0077 ± 0.0024
Small intestine	0.0055 ± 0.0006	0.0082 ± 0.0005	< 0.0001	0.0116 ± 0.0012	0.0078 ± 0.0025
Stomach wall	0.0053 ± 0.0007	0.0120 ± 0.0027	0.0008 ± 0.0002	0.0106 ± 0.0010	0.0077 ± 0.0023
Upper large intestine wall	0.0055 ± 0.0007	0.0075 ± 0.0004	0.0003 ± 0.0001	0.0113 ± 0.0011	0.0078 ± 0.0024
Heart wall	0.0111 ± 0.0013	0.0204 ± 0.0028	< 0.0001	0.0229 ± 0.0028	0.0134 ± 0.0049
Kidneys	0.0160 ± 0.0046	0.0324 ± 0.0072	0.0002 ± 0.0001	0.0294 ± 0.0079	0.0431 ± 0.0234
Liver	0.0101 ± 0.008	0.0145 ± 0.0036	0.0003 ± 0.0001	0.0150 ± 0.0036	0.0223 ± 0.0181
Lungs	0.0050 ± 0.0007	0.0233 ± 0.0044	0.0019 ± 0.0005	0.0096 ± 0.0007	0.0142 ± 0.0053
Muscle	0.0050 ± 0.0007			0.0094 ± 0.0010	
Ovaries	0.0058 ± 0.0007		0.0004 ± 0.0001	0.0125 ± 0.0016	0.0084 ± 0.0027
Pancreas	0.0057 ± 0.0008	0.0306 ± 0.0111	< 0.0001	0.0118 ± 0.0010	0.0084 ± 0.0027
Red marrow	0.0071 ± 0.001	0.0145 ± 0.0013	0.0008 ± 0.0001	0.0112 ± 0.0011	0.0060 ± 0.0019
Osteogenic cells	0.0094 ± 0.0013	0.0107 ± 0.0009		0.0153 ± 0.0014	0.0042 ± 0.0013
Spleen	0.0070 ± 0.0028	0.0225 ± 0.0095	< 0.0001	0.0167 ± 0.0044	0.0079 ± 0.0025
Testes	0.0049 ± 0.0007	0.0071 ± 0.0005	< 0.0002	0.0099 ± 0.0013	0.0073 ± 0.0025
Thymus	0.0051 ± 0.0006	0.0074 ± 0.0003	< 0.0001	0.0102 ± 0.0009	0.0075 ± 0.0023
Thyroid	0.0048 ± 0.0006	0.0331 ± 0.0074	0.0002 ± 0.0001	0.0091 ± 0.0009	0.0067 ± 0.0021
Urinary bladder wall	0.0483 ± 0.0086	0.2260 ± 0.0331	0.0058 ± 0.0071	0.0758 ± 0.0284	0.0998 ± 0.0687
Uterus	0.0095 ± 0.0054		0.0002 ± 0.0001	0.0149 ± 0.0025	0.0106 ± 0.0036
Total body	0.0058 ± 0.0012	0.0089 ± 0.0004	0.0127 ± 0.0074	0.0097 ± 0.0009	0.0082 ± 0.0025
Effective dose (mSv/MBq)	0.0078 ± 0.0013	0.0194 ± 0.0017	0.0127 ± 0.0067	0.0141 ± 0.0022	0.0116 ± 0.0047

## 9. WHAT DOES THE FIELD NEED

This document provides guidelines and recommendations based on the current available literature. FAP PET is in its early days, and there will be significant changes to our understanding of the role of FAP PET as we learn more, and this document will need to be updated. There are many unmet needs in the field. As FAP PET becomes used more frequently in the clinical setting, there needs to be a focus on reader training, especially given the numerous non-oncological lesions that have uptake (64). Possibly the most important strategy to move forward is to establish well designed prospective clinical trials which both help elucidate the clinical role of FAP PET, but also lead to regulatory approval of these imaging agents. Prospective trials will focus on staging, but there will also be a need to better understand the clinical impact of more accurate disease detection and treatment response assessment, as patients will receive follow-up FAP PET. FAP PET is an incredibly promising imaging agent, and we look forward to its broad future in clinical practice.

## LIABILITY STATEMENT

This guideline summarizes the views of the EANM Oncology & Theranostics Committee and the SNMMI. It reflects recommendations for which the EANM and SNMMI cannot be held responsible. The recommendations should be taken into context of good practice of nuclear medicine and do not substitute for national and international legal or regulatory provisions.

## References

[1] Park JE Lenter MC Zimmermann RN Garin-Chesa P Old LJ Rettig WJ. Fibroblast activation protein, a dual specificity serine protease expressed in reactive human tumor stromal fibroblasts. J Biol Chem. 1999;274:36505–36512. 10593948 10.1074/jbc.274.51.36505

[2] Juillerat-Jeanneret L Tafelmeyer P Golshayan D. Fibroblast activation protein-α in fibrogenic disorders and cancer: more than a prolyl-specific peptidase? Expert Opin Ther Targets. 2017;21:977–991. 28829211 10.1080/14728222.2017.1370455

[3] Kalluri R. The biology and function of fibroblasts in cancer. Nat Rev Cancer. 2016;16:582–598. 27550820 10.1038/nrc.2016.73

[4] Chen X Song E. Turning foes to friends: targeting cancer-associated fibroblasts. Nat Rev Drug Discov. 2019;18:99–115. 30470818 10.1038/s41573-018-0004-1

[5] Kratochwil C Flechsig P Lindner T. ^68^Ga-FAPI PET/CT: tracer uptake in 28 different kinds of cancer. J Nucl Med. 2019;60:801–805. 30954939 10.2967/jnumed.119.227967 PMC6581228

[6] Mosessian S Jensen JD Enke AS. Current state of clinical trials and regulatory approvals with fibroblast activation protein targeting interventions. PET Clin. 2023;18:429–439. 36990947 10.1016/j.cpet.2023.02.010

[7] Whatcott CJ Diep CH Jiang P. Desmoplasia in primary tumors and metastatic lesions of pancreatic cancer. Clin Cancer Res. 2015;21:3561–3568. 25695692 10.1158/1078-0432.CCR-14-1051 PMC4526394

[8] Wang L Tang G Hu K. Comparison of ^68^ Ga-FAPI and ^18^ F-FDG PET/CT in the evaluation of advanced lung cancer. Radiology. 2022;303:191–199. 34981976 10.1148/radiol.211424

[9] Backhaus P Burg MC Roll W. Simultaneous FAPI PET/MRI targeting the fibroblast-activation protein for breast cancer. Radiology. 2022;302:39–47. 34636633 10.1148/radiol.2021204677

[10] Elboga U Sahin E Kus T. Superiority of ^68^Ga-FAPI PET/CT scan in detecting additional lesions compared to ^18^FDG PET/CT scan in breast cancer. Ann Nucl Med. 2021;35:1321–1331. 34436740 10.1007/s12149-021-01672-x

[11] Jiang Y Wen B Li C. The performance of ^68^Ga-FAPI-04 PET/CT in head and neck squamous cell carcinoma: a prospective comparison with ^18^F-FDG PET/CT. Eur J Nucl Med Mol Imaging. 2023;50:2114–2126. 36808001 10.1007/s00259-023-06138-y

[12] Zheng W Liu L Feng Y Wang L Chen Y. Comparison of ^68^Ga-FAPI-04 and fluorine-18-fluorodeoxyglucose PET/computed tomography in the detection of ovarian malignancies. Nucl Med Commun. 2023;44:194–203. 36472415 10.1097/MNM.0000000000001653 PMC9907692

[13] Zhao L Pang Y Luo Z. Role of [^68^Ga]Ga-DOTA-FAPI-04 PET/CT in the evaluation of peritoneal carcinomatosis and comparison with [^18^F]-FDG PET/CT. Eur J Nucl Med Mol Imaging. 2021;48:1944–1955. 33415432 10.1007/s00259-020-05146-6

[14] Lanzafame H Mavroeidi IA Pabst KM. ^68^Ga-fibroblast activation protein inhibitor PET/CT improves detection of intermediate and low-grade sarcomas and identifies candidates for radiopharmaceutical therapy. J Nucl Med. 2024;65:880–887. 38724279 10.2967/jnumed.123.267248

[15] Li K Liu W Yu H. ^68^Ga-FAPI PET imaging monitors response to combined TGF-βR inhibition and immunotherapy in metastatic colorectal cancer. J Clin Invest. 2024;134:e170490. 38618961 10.1172/JCI181374 PMC11014646

[16] Chen L Zheng S Chen L. ^68^Ga-labeled fibroblast activation protein inhibitor PET/CT for the early and late prediction of pathologic response to neoadjuvant chemotherapy in breast cancer patients: a prospective study. J Nucl Med. 2023;64:1899–1905. 37918866 10.2967/jnumed.123.266079 PMC10690122

[17] Maliha PG Hotta M Farolfi A. FAPI PET uptake patterns after invasive medical interventions: a single center retrospective analysis. Eur J Nucl Med Mol Imaging. 2024;51:3373–3385. 38750372 10.1007/s00259-024-06733-7

[18] Mori Y Dendl K Cardinale J Kratochwil C Giesel FL Haberkorn U. FAPI PET: fibroblast activation protein inhibitor use in oncologic and nononcologic disease. Radiology. 2023;306:e220749. 36594838 10.1148/radiol.220749

[19] Schmidkonz C. Perspective on fibroblast activation protein–specific PET/CT in fibrotic interstitial lung diseases: imaging fibrosis—a new paradigm for molecular imaging? J Nucl Med. 2022;63:125–126. 34649945 10.2967/jnumed.121.262944 PMC8717189

[20] Windisch P Zwahlen DR Giesel FL. Clinical results of fibroblast activation protein (FAP) specific PET for non-malignant indications: systematic review. EJNMMI Res. 2021;11:18. 33606104 10.1186/s13550-021-00761-2 PMC7895887

[21] Sviridenko A Di Santo G Virgolini I. Imaging fibrosis. PET Clin. 2023;18:381–388. 36990946 10.1016/j.cpet.2023.02.004

[22] Zhang M Quan W Zhu T. [^68^Ga]Ga-DOTA-FAPI-04 PET/MR in patients with acute myocardial infarction: potential role of predicting left ventricular remodeling. Eur J Nucl Med Mol Imaging. 2023;50:839–848. 36326870 10.1007/s00259-022-06015-0 PMC9852131

[23] Notohamiprodjo S Nekolla SG Robu S. Imaging of cardiac fibroblast activation in a patient after acute myocardial infarction using 68Ga-FAPI-04. J Nucl Cardiol. 2022;29:2254–2261. 33860458 10.1007/s12350-021-02603-z PMC9553764

[24] Diekmann J Koenig T Thackeray JT. Cardiac fibroblast activation in patients early after acute myocardial infarction: integration with MR tissue characterization and subsequent functional outcome. J Nucl Med. 2022;63:1415–1423. 35210301 10.2967/jnumed.121.263555 PMC9454470

[25] Barton AK Tzolos E Bing R. Emerging molecular imaging targets and tools for myocardial fibrosis detection. Eur Heart J Cardiovasc Imaging. 2023;24:261–275. 36575058 10.1093/ehjci/jeac242 PMC9936837

[26] Yang P Luo Q Wang X. Comprehensive analysis of fibroblast activation protein expression in interstitial lung diseases. Am J Respir Crit Care Med. 2023;207:160–172. 35984444 10.1164/rccm.202110-2414OC PMC9893314

[27] Röhrich M Leitz D Glatting FM. Fibroblast activation protein–specific PET/CT imaging in fibrotic interstitial lung diseases and lung cancer: a translational exploratory study. J Nucl Med. 2022;63:127–133. 34272325 10.2967/jnumed.121.261925 PMC8717194

[28] Scott AM Wiseman G Welt S. A phase I dose-escalation study of sibrotuzumab in patients with advanced or metastatic fibroblast activation protein-positive cancer. Clin Cancer Res. 2003;9:1639–1647. 12738716

[29] Meany H Balis FM Aikin A. Pediatric phase I trial design using maximum target inhibition as the primary endpoint. J Natl Cancer Inst. 2010;102:909–912. 20460632 10.1093/jnci/djq174 PMC2886096

[30] Bergmann C Distler JHW Treutlein C. ^68^Ga-FAPI-04 PET-CT for molecular assessment of fibroblast activation and risk evaluation in systemic sclerosis-associated interstitial lung disease: a single-centre, pilot study. Lancet Rheumatol. 2021;3:e185–e194. 38279381 10.1016/S2665-9913(20)30421-5

[31] Zhang Z Yu Y Zhang L Cheng C Zuo C. ^18^F-FDG and ^68^Ga-FAPI-04 in the evaluation of aggressive perivascular epithelioid cell tumor. Clin Nucl Med. 2022;47:897–899. 35485860 10.1097/RLU.0000000000004249

[32] Assadi M Rekabpour SJ Jafari E. Feasibility and therapeutic potential of ^177^Lu-fibroblast activation protein inhibitor-46 for patients with relapsed or refractory cancers: a preliminary study. Clin Nucl Med. 2021;46:e523–e530. 34269729 10.1097/RLU.0000000000003810

[33] Backhaus P Gierse F Burg MC. Translational imaging of the fibroblast activation protein (FAP) using the new ligand [^68^Ga]Ga-OncoFAP-DOTAGA. Eur J Nucl Med Mol Imaging. 2022;49:1822–1832. 34957527 10.1007/s00259-021-05653-0 PMC9016025

[34] Millul J Bassi G Mock J. An ultra-high-affinity small organic ligand of fibroblast activation protein for tumor-targeting applications. Proc Natl Acad Sci USA. 2021;118:e2101852118. 33850024 10.1073/pnas.2101852118 PMC8072232

[35] Zhao L Niu B Fang J. Synthesis, preclinical evaluation, and a pilot clinical PET imaging study of ^68^ Ga-labeled FAPI dimer. J Nucl Med. 2022;63:862–868. 34556528 10.2967/jnumed.121.263016 PMC9157726

[36] Lindner T Loktev A Altmann A. Development of quinoline-based theranostic ligands for the targeting of fibroblast activation protein. J Nucl Med. 2018;59:1415–1422. 29626119 10.2967/jnumed.118.210443

[37] Zboralski D Hoehne A Bredenbeck A. Preclinical evaluation of FAP-2286 for fibroblast activation protein targeted radionuclide imaging and therapy. Eur J Nucl Med Mol Imaging. 2022;49:3651–3667. 35608703 10.1007/s00259-022-05842-5 PMC9399058

[38] Pang Y Zhao L Meng T. PET imaging of fibroblast activation protein in various types of cancer using ^68^Ga-FAP-2286: comparison with ^18^F-FDG and ^68^Ga-FAPI-46 in a single-center, prospective study. J Nucl Med. 2023;64:386–394. 36215571 10.2967/jnumed.122.264544 PMC10071807

[39] Kline B Yadav S Seo Y. ^68^Ga-FAP-2286 PET of solid tumors: biodistribution, dosimetry, and comparison with ^18^F-FDG. J Nucl Med. 2024;65:938–943. 38697672 10.2967/jnumed.123.267281 PMC11149593

[40] Chen Z Wang Y Yang X. Feasibility of acquisitions using total-body PET/CT with a half-dose [^68^Ga]Ga-FAPI-04 activity in oncology patients. Eur J Nucl Med Mol Imaging. 2023;50:3961–3969. 37535107 10.1007/s00259-023-06354-6

[41] Glatting FM Hoppner J Liew DP. Repetitive early ^68^Ga-FAPI PET acquisition comparing ^68^Ga-FAPI-02, ^68^Ga-FAPI-46, and ^68^Ga-FAPI-74: methodologic and diagnostic implications for malignant, inflammatory/reactive, and degenerative lesions. J Nucl Med. 2022;63:1844–1851. 35618480 10.2967/jnumed.122.264069 PMC9730916

[42] Hu K Wang L Wu H. [^18^F]FAPI-42 PET imaging in cancer patients: optimal acquisition time, biodistribution, and comparison with [^68^Ga]Ga-FAPI-04. Eur J Nucl Med Mol Imaging. 2022;49:2833–2843. 34893920 10.1007/s00259-021-05646-z

[43] Naeimi M Choyke PL Dendl K. Three-time-point PET analysis of ^68^ Ga-FAPI-46 in a variety of cancers. J Nucl Med. 2023;64:618–622. 36357183 10.2967/jnumed.122.264941 PMC11927082

[44] Röhrich M Naumann P Giesel FL. Impact of ^68^Ga-FAPI PET/CT imaging on the therapeutic management of primary and recurrent pancreatic ductal adenocarcinomas. J Nucl Med. 2021;62:779–786. 33097632 10.2967/jnumed.120.253062 PMC8729866

[45] Giesel FL Adeberg S Syed M. FAPI-74 PET/CT using either ^18^F-AlF or cold-kit ^68^Ga labeling: biodistribution, radiation dosimetry, and tumor delineation in lung cancer patients. J Nucl Med. 2021;62:201–207. 32591493 10.2967/jnumed.120.245084 PMC8679591

[46] Nadig V Herrmann K Mottaghy FM Schulz V. Hybrid total-body pet scanners—current status and future perspectives. Eur J Nucl Med Mol Imaging. 2022;49:445–459. 34647154 10.1007/s00259-021-05536-4 PMC8803785

[47] van Sluis J Van Snick JH Brouwers AH. EARL compliance and imaging optimisation on the Biograph Vision Quadra PET/CT using phantom and clinical data. Eur J Nucl Med Mol Imaging. 2022;49:4652–4660. 35876867 10.1007/s00259-022-05919-1 PMC9606094

[48] Wielaard J Habraken JBA Brinks P Lavalaye J Boellaard R. Optimization of injected ^68^Ga-PSMA activity based on list-mode phantom data and clinical validation. EJNMMI Phys. 2020;7:20. 32297142 10.1186/s40658-020-00289-9 PMC7158971

[49] Giesel FL Kratochwil C Lindner T. ^68^Ga-FAPI PET/CT: biodistribution and preliminary dosimetry estimate of 2 DOTA-containing FAP-targeting agents in patients with various cancers. J Nucl Med. 2019;60:386–392. 30072500 10.2967/jnumed.118.215913 PMC6424229

[50] Mona CE Benz MR Hikmat F. Correlation of ^68^Ga-FAPi-46 PET biodistribution with FAP expression by immunohistochemistry in patients with solid cancers: interim analysis of a prospective translational exploratory study. J Nucl Med. 2022;63:1021–1026. 34740953 10.2967/jnumed.121.262426 PMC9258565

[51] Gil J Choi H Paeng JC Kang KW Jang J-Y. Accumulation of [^18^F]FAPI-74 in biliary system as a potential pitfall in evaluating pancreaticobiliary cancer [abstract]. J Nucl Med. 2022;63(suppl 2):3034.

[52] Kilvaer TK Rakaee M Hellevik T. Tissue analyses reveal a potential immune-adjuvant function of FAP-1 positive fibroblasts in non-small cell lung cancer. Gullberg D, ed. PLoS ONE. 2018;13:e0192157. 29415055 10.1371/journal.pone.0192157 PMC5802915

[53] Kessler L Ferdinandus J Hirmas N. Pitfalls and common findings in ^68^ Ga-FAPI PET: a pictorial analysis. J Nucl Med. 2022;63:890–896. 34620730 10.2967/jnumed.121.262808 PMC9157730

[54] Hotta M Rieger AC Jafarvand MG. Non-oncologic incidental uptake on FAPI PET/CT imaging. Br J Radiol. 2023;96:20220463. 35776566 10.1259/bjr.20220463 PMC9975522

[55] Zheng S Lin R Chen S. Characterization of the benign lesions with increased ^68^Ga-FAPI-04 uptake in PET/CT. Ann Nucl Med. 2021;35:1312–1320. 34424505 10.1007/s12149-021-01673-w

[56] Zhang X Song W Qin C. Uterine uptake of ^68^Ga-FAPI-04 in uterine pathology and physiology. Clin Nucl Med. 2022;47:7–13. 34874344 10.1097/RLU.0000000000003968

[57] Dendl K Koerber SA Watabe T Haberkorn U Giesel FL. Current status of fibroblast activation protein imaging in gynecologic malignancy and breast cancer. PET Clin. 2023;18:345–351. 37257985 10.1016/j.cpet.2023.03.005

[58] Pan Q Luo Y Zhang W. Idiopathic retroperitoneal fibrosis with intense uptake of ^68^Ga-fibroblast activation protein inhibitor and ^18^F-FDG. Clin Nucl Med. 2021;46:175–176. 33208623 10.1097/RLU.0000000000003402

[59] Koerber SA. Radiation therapy planning using fibroblast activation protein inhibitor. PET Clin. 2023;18:369–380. 37117122 10.1016/j.cpet.2023.03.003

[60] Chen H Pang Y Wu J. Comparison of [^68^Ga]Ga-DOTA-FAPI-04 and [^18^F] FDG PET/CT for the diagnosis of primary and metastatic lesions in patients with various types of cancer. Eur J Nucl Med Mol Imaging. 2020;47:1820–1832. 32222810 10.1007/s00259-020-04769-z

[61] Ding J Qiu J Hao Z. Comparing the clinical value of baseline [^68^Ga]Ga-FAPI-04 PET/CT and [^18^F]F-FDG PET/CT in pancreatic ductal adenocarcinoma: additional prognostic value of the distal pancreatitis. Eur J Nucl Med Mol Imaging. 2023;50:4036–4050. 37493664 10.1007/s00259-023-06297-y

[62] Zhao L Pang Y Sun L Lin Q Wu H Chen H. Fibroblast activation protein inhibitor PET in pancreatic cancer. PET Clin. 2023;18:295–308. 37030983 10.1016/j.cpet.2023.02.001

[63] Röhrich M Daum J Gutjahr E. Diagnostic potential of supplemental static and dynamic ^68^Ga-FAPI-46 PET for primary ^18^F-FDG–negative pulmonary lesions. J Nucl Med. 2024;65:872–879. 38604763 10.2967/jnumed.123.267103 PMC11149599

[64] Mei R Kessler L Pabst KM. ^68^Ga-FAPI PET/CT interobserver agreement on tumor assessment: an international multicenter prospective study. J Nucl Med. 2023;64:1043–1048. 37230530 10.2967/jnumed.122.265245

